# Precision Medicine Targeting *FGFR2* Genomic Alterations in Advanced Cholangiocarcinoma: Current State and Future Perspectives

**DOI:** 10.3389/fonc.2022.860453

**Published:** 2022-04-04

**Authors:** Miguel Zugman, Gehan Botrus, Roberto Carmagnani Pestana, Pedro Luiz Serrano Uson Junior

**Affiliations:** ^1^Department of Oncology, Hospital Israelita Albert Einstein, São Paulo, Brazil; ^2^Medical Oncology Phase I Clinical Trials, HonorHealth Research Institute, Phoenix, AZ, United States; ^3^Center for Personalized Medicine, Hospital Israelita Albert Einstein, São Paulo, Brazil

**Keywords:** cholangiocarcinoma, FGFR2, fusions, precision medicine, pemigatinib

## Abstract

Although a relatively uncommon tumor, cholangiocarcinoma is on the rise globally. Of note, most patients are diagnosed with metastatic disease, and the prognosis is poor with cytotoxic chemotherapy. Strategies targeting specific genomic alterations have demonstrated promising activity in recent years and could represent a new therapeutic avenue for these patients. In this review, we will address the biology and clinical results of FGFR inhibition in intrahepatic cholangiocarcinoma, highlighting limitations associated with treatment and discussing the use of circulating tumor DNA to detect mechanisms of resistance.

## Introduction

Biliary tract cancers (BTCs) are a group of heterogenous and rare malignancies that arise from any point of the biliary tract yet are uniformly associated with poor prognosis. BTCs are subdivided in intrahepatic cholangiocarcinoma (ICCA), extrahepatic cholangiocarcinoma (ECCA) and gallbladder cancer. ICCA originates from within the liver parenchyma, whereas ECCA can arise from any portion of the biliary tract outside of the liver, which can be further classified as hilar or distal cholangiocarcinoma (1). Incidence worldwide is increasing, both from ICCA and ECCA (2, 3). The estimated number of new cases of primary liver cancer, including hepatocellular carcinoma and biliary cancers, to have occurred globally in 2020, were of 906.000, of which ICCA accounts for approximately 10-15% (4).

The therapy of choice for advanced BTCs was established by the ABC-02 phase III trial, OS was significantly improved with gemcitabine and cisplatin versus gemcitabine (median 11.7 versus 8.1 months, HR 0.64) (5, 6). A phase II, non-randomized, single-arm clinical trial investigated the addition of nab-paclitaxel to gemcitabine-cisplatin (7). Median PFS was 12.2 months, and median OS was 19.2 months, which compares favorably to historical controls. Lately, positive results with the addition of durvalumab to chemotherapy was achieved in the TOPAZ-1 trial (8). In the study, durvalumab combined with cisplatin and gemcitabine conferred a 20% reduction in the risk of death compared with gemcitabine and cisplatin alone, meeting the primary endpoint of the trial, PFS and response rate were also improved with the combination (8). Although FOLFIRINOX is an effective regimen in pancreatic cancer, in advanced biliary cancers it was not superior to gemcitabine and cisplatin in the phase II randomized trial PRODIGE 38 AMEBICA (9). For second-line chemotherapy, results are less encouraging. Randomized trials identified mFOLFOX or 5FU plus liposomal irinotecan as regimens considered second-line options with improvements in OS for patients who have progressed after gemcitabine-based treatment, although more efficacious treatments are in need (10, 11).

**Figure 1:**
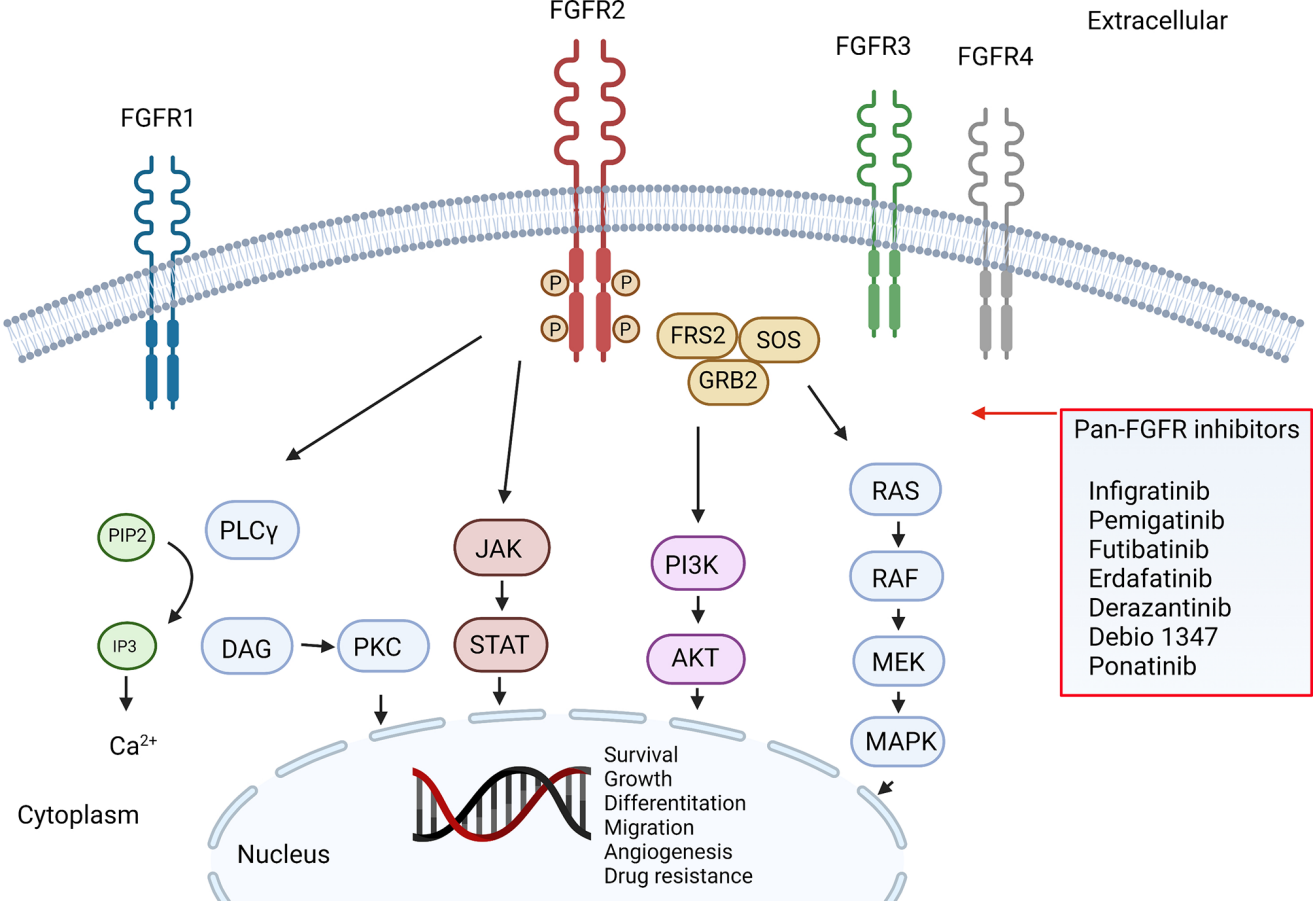
FGFR pathway and inhibitors.

Biomarkers are present in varying patterns among ICCA and ECCA, and such differences highlight tumor-specific oncogenic pathways (12). Some of these biomarkers predicted the response to fibroblast growth factor receptor (*FGFR*) inhibitors, which target *FGFR2*-fusions in ICCA (**Table 1**, **Figure 1**). The *FGFR2* belongs to the *FGFR* family of tyrosine kinases receptors. The family consists of 4 genes that encode single-pass transmembrane receptors that bind to *FGF* on the extracellular domain. Ligand binding trigger a signaling cascade that may exercise several cellular functions, including cell survival (13). It is estimated that *FGFR2* genomic alterations are present in around 10-15% of ICCA, most of them consisting of fusions (14), but also different aberrations can drive oncogenic transformation, such as mutations and amplifications, which may account for up to 3% of the cases (15).

**Table 1:** *FGFRi*s of interest in cholangiocarcinoma with *FGFR2* genomic alterations.

Drug	Mechanism of action	Design of study	N°	FGFR alteration	Patient characteristics	Response rate (95%CI)	Median Progression-free survival months (95%CI)	Median Overall survival months (95%CI)
Pemigatinib	*FGFR1-3* inhibitor	Phase II	146	107 *FGFR2* fusions/rearrangements	≥ 1 previous line of systemic treatment	35.5% (26.5-45.4)	6.9 (6.2-9.6)	21.1 (14.8-NE)
				20 other *FGF/FGFR* alterations		0	2.1 (1.2-4.2)	6.7 (2.1-10.6)
				18 no *FGF/FGFR* alteration			1.7 (1.3-1.8)	4 (2.3-6.5)
Infigratinib	*FGFR1-3* inhibitor	Phase II	108	83 *FGFR2* fusions/rearrangements	≥ 1 previous line of systemic treatment	23.1% (15.6-32.2)	7.3 (5.6-7.6)	Not reached
Futibatinib*	*FGFR1-4* inhibitor	Phase II	67	*FGFR2* fusions/rearrangements	≥ 1 previous line of systemic treatment	37.3% (-)	–	–
Derazantinib	*FGFR1-3* inhibitor	Phase I/II	29	*FGFR2* fusions/rearrangements	≥ 1 previous line of systemic treatment or ineligible for chemotherapy	20.7%	5.7 (4.0-9.2)	Not reached
Erdafatinib	*FGFR1-4* inhibitor	Phase II	10	*FGFR2* fusions or mutations	≥ 1 previous line of systemic treatment	60%	12.3 (3.1-19.3)	–
Debio 1347	*FGFR1-3* inhibitor	Phase I	5	*FGFR1-3* fusions	≥ 1 previous line of systemic treatment	40%	–	–
Ponatinib	*FGFR1-4* inhibitor	Phase I	12	*FGFR* genomic alterations	≥ 1 previous line of systemic treatment	9.1% (0.2-41.3)	–	–

NE, Not estimable; N°, number of patients; * irreversible.

Chromosomal rearrangements (i.e., Fusions) cause intragenic translocations that encode functional proteins derived from each of the original proteins. *FGFR2* partners up with other proteins with strong dimerization capacity, resulting in constitutive receptor activation and downstream signaling (16). Normally, FGF-FGFR signaling is triggered by the ligand-dependent receptor dimerization. The activation of the receptor leads to intracellular phosphorylation of receptor kinase domains, a cascade of intracellular signaling, and gene transcription. *FGFR2* constitutive kinase activity is linked to oncogene addictive pathways including the *RAS-MAPK, JAK-STAT*, and *PIK3-AKT-mTOR*, promoting progressive growth, invasiveness, epithelial-mesenchymal transition and neo angiogenesis, **Figure 1** (17). Single point mutations have been shown as well to increase *FGFR* activity by enhancing ligand binding of altering ligand specificity, they can also impair autoinhibitory brakes, which eventually turn to constitutive activity of the receptor kinase domain (18). All this alterations in FGFR genes, including activating mutations, chromosomal translocations, gene fusions, and gene amplifications, can result in ligand-independent signaling which increase receptor kinase activity.

## Pemigatinib

Pemigatinib is a tyrosine multi-kinase inhibitor that blocks FGFR1-3, with weaker activity against FGFR4 [Merz, Valeria, Camilla Zecchetto, and Davide Melisi. “Pemigatinib, a potent inhibitor of FGFRs for the treatment of cholangiocarcinoma.” Future Oncology 17.4 (2020): 389-402]. It has been shown that pemigatinib inhibits the growth of tumor cell lines in pre-clinical models, suppressing growth of xenografted tumor models with FGFR alterations (19).

Initially, pemigatinib was evaluated in a phase I/II open-label study in a subset of patients with advanced solid tumors (FIGHT-101) (20). In this study, pemigatinib was evaluated in three subsets of patients. Groups 1 and 3 had unselected advanced solid tumors and group 2 tumors harboring *FGF/FGFR* alteration. All patients with advanced solid tumors were refractory to prior therapy and had no further effective standard therapy.

Patients received pemigatinib orally once daily on a 21-day cycle (2-weeks on/1-week off). In the dose escalation group 1, first 3 cohorts (1-4 mg once daily) evaluated single patients and subsequently a 3 + 3 design was used (6-20 mg once daily). In the dose expansion group 2, patients with *FGFR* rearrangements started on 9 mg once daily and increased to 13.5 mg once daily. In part 3 (dose-finding and expansion) pemigatinib could be used in together with standard systemic therapies. Overall, about half of the patients presented hyperphosphatemia and fatigue, other adverse events observed included dry mouth, alopecia and stomatitis; most frequent grade ≥3 adverse events were pneumonia (10%), fatigue (8%), and hyponatremia (8%). Hyperphosphatemia was easily managed with diet and phosphate binders; further dose modifications was also necessary. Based on preliminary safety and efficacy, the recommended phase II dose selected was 13.5 mg once daily. In the dose expansion cohort, group 2, four patients with cholangiocarcinoma were treated with pemigatinib, with one achieving a partial response (PR) taking 9mg daily, with duration of response still ongoing at data cut-off (20).

The efficacy of pemigatinib in cholangiocarcinoma harboring *FGFR* alterations was further evaluated in the phase II study FIGHT-202 (21). Patients with cholangiocarcinoma and disease progression after at least one previous treatment were assigned to one of three cohorts: patients with *FGFR2*-fusions or rearrangements, patients with other *FGFR* alterations, or patients with no *FGFR* alterations. The primary endpoint was objective response rate (ORR) among those with fusions or rearrangements. From 1206 patients prescreened, a total of 146 patients were enrolled from multiple centers in USA, Europe, Middle East, and Asia; 107 patients had cholangiocarcinoma harboring fusions or rearrangements, 20 harbored other *FGF/FGFR* alterations, and 18 had no *FGF/FGFR* alterations. All patients received at least one dose of pemigatinib. After a median follow-up of 17.8 months, an ORR of 35.5% was observed in the 107 patients with fusions or rearrangements. Median PFS was 6.9 months and median OS was 21.1 months. No patients with other *FGF/FGFR* alterations achieved responses. The most common all-grade adverse event was hyperphosphatemia in 60% patients. Most frequent grade ≥3 adverse events were hypophosphatemia, arthralgia, stomatitis, hyponatremia, abdominal pain, and fatigue. Most frequent serious adverse events were abdominal pain, pyrexia, cholangitis, and pleural effusion. There were no treatment related deaths (21). Additionally, in another study, genomic analysis of patients who progressed on pemigatinib also revealed important information about this treatment (15). No statistical difference was observed in RR and PFS between cases classified as *FGFR2*-fusion versus rearranged (15). However, patients with co-occurring tumor suppression gene loss (e.g., *BAP1, CDKN2A/B, TP53, PBRM1, ARID1A*, or *PTEN*) had shorter median PFS (p= 0.0003) (15).

Hyperphosphatemia is one of the most common adverse events related to FGFR inhibitors. It is an on-target effect related to FGFR inhibition [Kommalapati, Anuhya, et al. “FGFR inhibitors in oncology: insight on the management of toxicities in clinical practice.” *Cancers* 13.12 (2021): 2968]. Multiple strategies are proposed to manage or prevent this adverse event, which includes dietary modifications, phosphate-lowering therapies classified into phosphate binders and phosphaturic agents and dose or schedule modifications. Available phosphate binders include magnesium hydroxide, calcium and iron-based regimens, lanthanum carbonate, and sevelamer. A phosphaturic agent commonly used is acetazolamide [Kommalapati, Anuhya, et al. “FGFR inhibitors in oncology: insight on the management of toxicities in clinical practice.” *Cancers* 13.12 (2021): 2968].

On April 2020, the U.S. Food and Drug Administration (FDA) approved pemigatinib for the treatment of patients with previously treated advanced cholangiocarcinoma with *FGFR2*-fusion or rearrangement. Pemigatinib was also approved in the same terms by European Commission on March 2021. Currently, an international phase III randomized trial is recruiting patients to address pemigatinib against platinum-based chemotherapy as first-line therapy for unresectable or metastatic cholangiocarcinoma with *FGFR2*-fusions or rearrangements (22).

## Infigratinib

The *FGFR* inhibitor (FGFRi) infigratinib was prospectively evaluated in patients with advanced cholangiocarcinoma with *FGFR* genomic alterations. In this phase II study, a total of 61 patients were evaluated and treated with infigratinib 125 mg orally for 21 days of each 28-day cycle until unacceptable toxicity or disease progression. All patients were previously treated with chemotherapy, including 67% of patients with at least two previous treatments. Most patients had cholangiocarcinoma harboring fusions, (n=48, 78.7%), eight patients had *FGFR* mutations, and three patients had amplifications (23). Eleven (18%) patients were treated previously with 3 lines of systemic therapy, and 19.7% with 4 lines. RR of patients harboring fusions was 18.8% [23. Overall DCR was 75.4% with a median PFS of 5.8 months. Updated results of the study were presented in a cohort of 108 patients, with 83 harboring *FGFR2*-fusions (24). In this subgroup, overall RR was 23.1%. A numerically higher RR was observed in patients treated in the second-line setting, RR of 34% (17/50), as compared with patients treated in the third or later-lines of systemic treatment (13.8%, 8/58). The median PFS of the cohort with fusions was 7.3 months. This result suggests that the efficacy of *FGFR2* inhibitors may be higher in earlier lines of systemic treatment for advanced cholangiocarcinoma; ergo, studies evaluating these drugs on first-line setting might demonstrate higher benefit of these drugs (24). Common adverse events (any grade) included hyperphosphatemia in 76.9%, in more than half of patients were observed eye disorders and stomatitis, fatigue was also common, in about 40% of patients treated. It is important to state that in this study all patients received prophylaxis with the oral phosphate binder sevelamer. On May 2021, based on these results, the FDA granted accelerated approval for infigratinib for the treatment of patients with previously treated advanced cholangiocarcinoma with an *FGFR2*-fusion or rearrangement. A randomized phase III trial of infigratinib versus gemcitabine plus cisplatin chemotherapy as first-line therapy for unresectable or metastatic cholangiocarcinoma with *FGFR2*-fusions or rearrangements is underway (25).

## Futibatinib

Futibatinib is an oral, highly selective, *FGFR1-4* irreversible inhibitor, unlike other drugs of this class that work by competitive antagonism. Futibatinib has first been tested in humans in a phase I dose-escalation study (FOENIX-101) which included 86 highly pretreated patients with diverse advanced solid tumors harboring *FGFR* aberrations. The study identified the 20mg daily dose as the recommended phase 2 dose. Five patients (5,8%) had an objective response, in which 3 patients had ICCA with *FGFR2*-fusions (26). Futibatinib was further tested in a dose expansion phase on 45 patients with cholangiocarcinoma (41 with ICCA) harboring *FGFR2*-aberrations, 28 of them (62%) FGFR2-fusions and 17 (38%) other *FGF-FGFR* aberrations. All patients had an ECOG score of 0 or 1 and had received prior systemic therapy, including 13 patients who had received at least one reversible *FGFRi*. Of the 28 patients with *FGFR2-*fusions, seven achieved confirmed PR (25%) and 15 patients (54%) had stable disease (SD). Four confirmed PR occurred in patients previously treated with *FGFRi*s, 3 of them with *FGFR2*-fusions (27). Grade 3 TRAE occurred in 41 patients (48%) of the overall population, the most frequent of them were hyperphosphatemia (12%), hyponatremia (7%) and anemia (6%). Other frequent any grade treatment emergent adverse events were diarrhea (37%), constipation (34%), dry mouth (29%), nausea (29%), and anemia (26%). Nail and ocular toxicity were also common.

That futibatinib achieves objective and durable responses after acquired secondary resistance to other *FGFRi*s may be attributable to its mechanism of action of covalent irreversible binding, permanently deactivating *FGFR2* enzymatic activity. Additional translational studies demonstrated futibatinib impressive capacity of overcoming diverse secondary *FGFR2* kinase domain mutations, providing evidence of benefit for serial biopsies and/or ctDNA analysis after treatment failure to identify potential strategies to overcome treatment resistance (28).

Futibatinib was further tested in the phase II, open-label, multicenter FOENIX-CCA2 trial in patients with locally advanced or metastatic cholangiocarcinoma with *FGFR2*-fusions or other rearrangements who have progressive disease (PD) after at least 1 systemic line of therapy, with no prior use of inhibitors. Interim results have been presented for 67 patients who were followed for at least 6 months. Most (82%) had *FGFR2*-fusions, and 18% other rearrangements. The ORR was 37.3%, median duration of response of 8.3 months and disease control rate of 82% for the overall population (29). Additionally, the phase III, open-label, randomized, FOENIX-CCA3 trial is currently recruiting patients to evaluate futibatinib efficacy versus gemcitabine-cisplatin chemotherapy in the treatment of advanced or recurrent ICCA harboring *FGFR2* gene rearrangements in the first-line setting (NCT04093362).

## Derazantinib

Derazantinib is a multi-kinase competitive inhibitor with potent activity against *FGFR1-3*. It was first tested in a phase I trial of an unselected patient population with advanced solid tumors (30). The study defined the 300mg daily dose as the recommended phase 2 dose. In this population, there were ten patients with ICCA, of which five harbored *FGFR2*-fusions. Two of these patients showed partial and durable responses, while one patient showed SD. These results prompted enrolment to a second part of the phase 1/2 trial of patients with *FGFR2*-fusion positive metastatic or inoperable ICCA, who had either progressed after at least one line of treatment or were ineligible for chemotherapy. Six (20.7%) achieved PR, 18 (62.1%) SD and five patients (17.2%) had PD. The median duration of disease control on those patients who had either response or SD was 5.8 months and the median PFS was 5.7 months. Median OS was not reached after a median follow up of 20 months. Adverse events were common, and treatment discontinuation occurred with four patients because of upper gastrointestinal bleeding. Hyperphosphatemia was reported in 22 patients but required no dose adjustment or interruption. Eye toxicity occurred in 12 patients (41.4%) which demanded dose interruption and/or reduction in seven patients (24.1%) (31). These comprised data led to the currently enrolling FIDES-01 trial, a phase 2 open-label, single-arm trial testing 300mg daily of derazantinib for patients with ICCA that harbor *FGFR2*-fusions, but also mutations or amplifications, as it was recently shown that derazantinib has similar efficacy in these cases (32).

## Erdafitinib

Erdafitinib is a potent, oral, *FGFR1-4* tyrosine kinase competitive inhibitor. A four-step phase I clinical trial assessed erdafitinib’s safety and tolerability, first in an unselected patient population and subsequently in patients with *FGFR* alterations, such as *FGFR3* mutations in urothelial carcinoma and *FGFR2*-fusions in ICCA (33). Although 187 patients were included, only 11 patients had ICCA, of which eight harbored *FGFR2*-fusions and three with *FGFR* mutations. In the overall cholangiocarcinoma population, 3 of 11 (27%) patients had PR, with a median duration of response of 11,4 months. The most common adverse events were hyperphosphatemia (64%), dry mouth (42%), and stomatitis, most of them of grade 1/2 severity. Skin changes, nail disorders, and eye disorders were also common. Grade 3 events or higher were infrequent, the main one reported was anemia in 17 patients (9%). Adverse events were considered the main cause of death in 9 patients (5%), including two cases of bleeding complications. Nevertheless, adverse events were mostly mild and manageable.

Erdafitinib is under further investigation in the LUC2001 trial, a phase II multi-center, open-label, clinical trial. Preliminary results of an Asian cohort of this study have been presented (34). Thirty-four patients with cholangiocarcinoma were found to harbor *FGFR* gene alterations and 14 of them were evaluated and treated with erdafitinib 8mg daily, with possible dose increases. Of these 14 patients, 8 had *FGFR2-*fusion ICCA while the remaining patients had different alterations and all patients had been previously treated with chemotherapy. In 10 evaluable patients with *FGFR2* alterations (gene fusion or mutation), there were 6 (60%) confirmed PR and 4 (40%) SD. Median PFS was 12.35 months. Safety and tolerability data were like those previously reported and did not differ in the Asian population compared to other ethnic groups.

## Debio-1347

Debio-1347 is an oral highly selective ATP competitive *FGFR1-3* inhibitor. The first-in-human study with the compound selected 58 patients with *FGFR1-3* alterations and defined 80mg daily as the standard dose (35). Efficacy was encouraging and safety analysis showed a manageable toxicity profile, with no deaths related to treatment. In the dose expansion phase, of the 18 patients enrolled for evaluable response, 5 had cholangiocarcinoma, of which 4 harbored *FGFR2*-fusion and one with *FGFR1*-fusion, being the only one who had PD after treatment. The other 4 had controlled disease, two with PR and two with SD. In total, 3 of the 18 patients had objective responses, with median duration of response of 16.1 weeks (range: 8.4-22.8) and median PFS of 18,3 weeks (36). The FUZE phase II basket-trial will further evaluate debio-1347 efficacy and tolerability in patients with solid tumors harboring *FGFR1-3* gene fusions previously treated and recruitment has already been completed (NCT03834220).

## Ponatinib

Ponatinib is a *FGFR1-4* tyrosine kinase inhibitor, along with inhibition effect in several other kinases including *KIT, RET, SRC, VEGFR* and *PDGFR* (37). A pilot study evaluating ponatinib in biliary tract cancers refractory to systemic treatments and *FGFR* alterations was early terminated after interim analysis (38). Overall disease control rate was 45.5% however objectives responses was observed in just one from eleven patients treated and assessed. Considering the modest activity of this agent futures studies should evaluate combinations with other molecules or refining patient selection (38).

## Discussion

*FGFR2*-fusions are an important target in cholangiocarcinoma to date; however, after an initially higher RR to these agents, most tumors will develop disease progression. Efforts are under way to identify mechanisms of resistance to this agents (15, 28, 39–41). Tumor resistance to *FGFRi*s is identified in multiple tumor types and are mostly related to activation of different signaling pathways including *MET*, *Eph3B, ERBB2/3* or *EGFR* and/or activation of intracellular signaling pathways without tyrosine kinase receptor dependence (42). Another observed factor of resistance with chronic exposure to *FGFRi*s is induced epithelial-mesenquimal transition (39). In cholangiocarcinoma, gatekeeper mutations that modify the binding site of *FGFRi*s and maintain activation of the *FGF* pathway have been described (39).

A sample from a patient with advanced *FGFR2* fused cholangiocarcinoma, harboring *FGFR2-CLIP1* fusion, was evaluated after progression to pemigatinib (40). Sanger sequencing on tumor samples after progression confirmed *FGFR2-CLIP1* fusion in all samples, furthermore, whole-exome sequencing revealed 242 unique mutations to post progression and a *FGFR2* kinase domain acquired mutation, *FGFR2* N549H in a single liver tumor. The *FGFR2 N549H* mutation results in ligand-independent constitutive activation (40).

Analysis with circulating tumor DNA (ctDNA) is also a strategy to identify acquired mutations (40). ctDNA analysis in three patients with advanced cholangiocarcinoma who disease progressed after treatment with infigratinib, identified multiple mutations on *FGFR* kinase domain including *N549H, N549K, V564F, E565A, K659M, L617V* and *K641R*. Paired tissue analysis detected *PI3K/PTEN* pathway mutations in some samples (15, 41). In the study evaluating clinicogenomic analysis of pemigatinib-treated patients, they identified a total of 63 *FGFR2* rearrangement partners genes (15). The most common fusion partners included *BICC1* (27.9%), *KIAA1217* (3.6%), *TACC2* (2.9%), *CCDC6* (2.9%), and *AHCYL1* (2.9%). The second most frequent *FGFR2* rearrangement identified was *FGFR2*-N/A (9.3%). N/A refers to rearrangements that occur in *FGFR2* intron 17 or exon 18 fused to an intergenic region (15). Interestingly, no differences in RR and PFS were observed between different fusion partners (15). Eight patients that initially responded to pemigatinib and had disease progression were evaluated with genomic profiling in tissue or plasma (15). The evaluation showed that all patients presented at least one acquired *FGFR2* mutation, suggesting that mechanisms of resistance to *FGFRi*s are similar between the different available drugs (15).

Targeted sequencing of tumor DNA after progression could delineate combination strategies to overcome resistance. Upregulation of the PI3K/AKT/mTOR signaling pathway was observed in cell lines expressing acquired FGFR2 p.E565A after infigratinib treatment. Further exposure to mTOR inhibitors re-sensitized these cells to FGFR inhibition [Krook, Melanie A., et al. “Efficacy of FGFR inhibitors and combination therapies for acquired resistance in FGFR2-fusion cholangiocarcinoma.” *Molecular Cancer Therapeutics* 19.3 (2020): 847-857]. Another strategy to overcome resistance would be combined EGFR/FGFR inhibition. In a study evaluating patient-derived models of FGFR2-fusion-positive cholangiocarcinoma, inhibition of EGFR potentiated responses to FGFR inhibitors, durably suppressing MEK/ERK and mTOR signaling, increasing apoptosis, and causing marked tumor regressions *in vivo*. [Wu, Qibiao, et al. “EGFR inhibition potentiates FGFR inhibitor therapy and overcomes resistance in FGFR2 fusion-positive cholangiocarcinomaCombination Treatment in FGFR2-Fusion Cholangiocarcinoma.” *Cancer Discovery*.]

In conclusion, incorporation of circulating tumor DNA analysis or tissue genomic analysis after exposure and disease progression on *FGFRi*s have the potential to characterize and understand specific gatekeeper mutations related to resistance and treatment failure. Exploring co-occurring mutations will be also necessary, considering that they could influence the effectiveness on these targeted treatments.

## Author Contributions

MZ performed the writing, prepared the table. RP and GB in writing and reviewing. PU provided the input in writing and reviewing the paper. All authors contributed to the article and approved the submitted version.

## Conflict of Interest

The authors declare that the research was conducted in the absence of any commercial or financial relationships that could be construed as a potential conflict of interest.

## Publisher’s Note

All claims expressed in this article are solely those of the authors and do not necessarily represent those of their affiliated organizations, or those of the publisher, the editors and the reviewers. Any product that may be evaluated in this article, or claim that may be made by its manufacturer, is not guaranteed or endorsed by the publisher.
